# Supplementary material for: Active dendrites enable robust spiking computations despite timing jitter
Source: eLife. 2026 Jul 27;12:RP89629. doi: 10.7554/eLife.89629 (PMC13405625; doi:10.7554/eLife.89629)
Supplement: Peer review history

# Editor report: eLife Assessment

**DOI:** 10.7554/eLife.89629.3.sa0

**Authors:** Gordon J Berman

**Affiliations:** Emory University United States

**Keywords:** Compelling, Important

This **important** study develops and exploits novel ideas in dendritic integration and implements these ideas in a neural network. Historically, dendritic plateau potentials were thought to exist primarily for maintaining neurons in a depolarized state for 100s of milliseconds, but this study presents a new perspective that dendritic plateau potentials are equally effective in much shorter integration windows. The computational evidence supporting the article's claims is **compelling**.

# Referee report: Reviewer #2 (Public review)

**DOI:** 10.7554/eLife.89629.3.sa1

Summary.

Some forms of Artificial Intelligence (AI), particularly those based on artificial neural networks (ANNs), draw inspiration from biological brains and neurons. Understanding the functional repertoire and underlying logic of real neurons could, therefore, help improve ANNs. While the cell bodies and axons of neurons produce rapid, high-amplitude action potentials (~100 mV over ~2 ms), dendrites-constituting about 80% of neuronal membrane area-generate smaller but longer-lasting electrical signals, known as glutamate-mediated dendritic plateau potentials (~50 mV over >100 ms). The authors have designed artificial neurons capable of producing these dendritic plateau potentials and, through simulations, demonstrate that such prolonged dendritic signals reduce the negative effects of temporal jitter in real or artificial neural networks. Specifically, they show that in ANNs with neurons capable of dendritic plateau potentials, reliable sparse spiking computation can occur without the need for precise input synchronization. This means that despite fluctuations in network activity (such as delays in the brain circuit responses, for example), neurons can still link related network events. Thus, dendritic plateau potentials enable neurons to retain information longer, connecting events that are not exactly simultaneous. Interestingly, one of the indirect conclusions of the current study is that neurons equipped with dendritic plateau potentials may reduce the total number of cells (nodes, units) required to perform robust computations.

Strengths.

Most studies in neuroscience are descriptive, focusing on observations and measurements. Fewer tackle the more challenging task of explaining the rationale behind specific natural designs. This study does just that, addressing the fundamental problem of asynchrony in neural communication caused by conduction delays and noise. Given that neurons with short membrane time constants can integrate only nearly simultaneous inputs, the authors propose a solution: dendritic plateau potentials. These potentials, generated through glutamate-mediated depolarization within dendritic branches, effectively broaden the temporal integration window, allowing neurons to handle temporal jitter, variability, stochasticity, and maintain reliable computation. Thus, dendritic plateau potentials appear to be an adaptive feature evolved to support rapid, reliable CNS computations.

Weaknesses.

The authors have appropriately revised unsupported statements from previous versions, but the manuscript could benefit from examples of testable hypotheses derived from their findings. For example, what specific experimental questions could be investigated to validate these computational predictions? Providing concrete examples of potential experimental tests would make the work more accessible and actionable for experimentalists, assuming such experiments are feasible.

Additionally, many readers may lack a background in computational modeling or Artificial Neural Networks. To enhance accessibility, key terms and concepts should be explained at a level suitable for first-year graduate students, ensuring clarity for a broader audience.

# Author comment: Author response

**DOI:** 10.7554/eLife.89629.3.sa2

**Authors:** Thomas SJ Burger, Michael E Rule, Timothy O'Leary

**Affiliations:** University of Cambridge Cambridge United Kingdom; University of Cambridge Cambridge United Kingdom; University of Cambridge Cambridge United Kingdom

The following is the authors’ response to the original reviews.

**Reviewer #1 (Public Review):**

This is an elegant didactic exposition showing how dendritic plateau potentials can enable neurons to perform reliable 'binary' computations in the face of realistic spike time jitter in cortical networks. The authors make many good arguments, and the general concept underlying the paper is sound. A strength is their systematic progression from biophiysical to simplified models of single neurons, and their parallel investigation of spiking and binary neural networks, with training happening in the binary neural network.

**Reviewer #2 (Public Review):**

Summary:

Artificial intelligence (AI) could be useful in some applications and could help humankind. Some forms of AI work on the platform of artificial neural networks (ANN). ANNs are inspired by real brains and real neurons. Therefore understanding the repertoire and logic of real neurons could potentially improve AANs. Cell bodies of real neurons, and axons of real neurons, fire nerve impulses (nerve impulses are very brief ~2 ms, and very tall ~100 mV). Dendrites, which comprise ~80% of the total neuronal membrane (80% of the total neuronal apparatus) typically generate smaller (~50 mV amplitude) but much longer (~100 ms duration) electrical transients, called glutamate-mediated dendritic plateau potentials. The authors have built artificial neurons capable of generating such dendritic plateau potentials, and through computer simulations the authors concluded that long-lasting dendritic signals

(plateau potentials) reduce negative impact of temporal jitter occurring in real brain, or in

AANs. The authors showed that in AANs equipped with neurons whose dendrites are capable of generating local dendritic plateau potentials, the sparse, yet reliable spiking computations may not require precisely synchronized inputs. That means, the real world can impose notable fluctuations in the network activity and yet neurons could still recognize and pair the related network events. In the AANs equipped with dendritic plateaus, the computations are very robust even when inputs are only partially synchronized. In summary, dendritic plateau potentials endow neurons with ability to hold information longer and connect two events which did not happen at the same moment of time. Dendritic plateaus circumvent the negative impact, which the short membrane time constants arduously inflict on the action potential generation (in both real neurons and model neurons). Interestingly, one of the indirect conclusions of the current study is that neurons equipped with dendritic plateau potentials may reduce the total number of cells (nodes, units) required to perform robust computations.

Strengths:

The majority of published studies are descriptive in nature. Researchers report what they see or measure. A smaller number of studies embark on a more difficult task, which is to explain the logic and rationale of a particular natural design. The current study falls into that second category. The authors first recognize that conduction delays and noise make asynchrony unavoidable in communication between circuits in the real brain. This poses a fundamental problem for the integration of related inputs in real (noisy) world. Neurons with short membrane time constants can only integrate coincident inputs that arrive simultaneously within 2-3 ms of one another. Then the authors considered the role for dendritic plateau potentials. Glutamate-mediated depolarization events within individual dendritic branches, can remedy the situation by widening the integration time window of neurons. In summary, the authors recognized that one important feature of neurons, their dendrites, are built-in to solve the major problems of rapid signal processing: [1] temporal jitter, [2] variation, [3] stochasticity, and [4] reliability of computation. In one word, the dendritic plateau potentials have evolved in the central nervous systems to make rapid CNS computations robust.

Weaknesses:

The authors made some unsupported statements, which should either be deleted, or thoroughly defended in the manuscript. But first of all, the authors failed to bring this study to the readers who are not experts in computational modeling or Artificial Neural Networks. Critical terms (syntax) and ideas have not been explained. For example: [1] binary feature space? [2] 13 dimensions binary vectors? [3] the binary network could still cope with the loss of information due to the binarization of the continuous coordinates? [4] accurate summation?

**Recommendations for the authors:**

**Reviewer #1 (Recommendations For The Authors):**

However, I have a number of specific points, listed below, that should be addressed. Most of them are relatively minor, but the authors should especially address point 10, which is a major point, by redoing the simulations affected by the erroneous value of the time constant, and by remaking the relevant figures based on the new simulations.

Specific comments:

(1) 7f "This feature is conspicuous because it is an order of magnitude longer than unitary synaptic inputs and axonal spikes.": — It is an order of magnitude longer than AMPA receptor-mediated synaptic currents (EPSCs), but more similar in time course to synaptic potentials (EPSPs) whose decay is governed by the passive membrane time constant (about 10 to 20 ms in pyramidal neurons in vivo) and which determines the lifetime of the 'memory' of the neuron for synaptic inputs under conditions of subthreshold, non-spiking dendritic integration. The quoted sentence should be rewritten accordingly.

Following this suggestion, we have rewritten the sentence (l. 7) to: "This timescale is conspicuous, being many times longer than the fastest signalling processes in the nervous systems, including Excitatory Post-Synaptic Potentials (EPSPs) and axonal spikes."

(2) 16ff "This is especially relevant to integration of inputs during high conductance states that are prevalent in-vivo. In these states the effective time constant of the neuronal membrane is extremely short and varies substantially depending on synaptic drive [13, 34, 49].": — The time-averaged synaptic conductance driven by sensory input in vivo is much less high than implied by this statement (e.g. see Fig. 4 of Haider et al. 2013 https://www.nature.com/articles/nature11665), and reduces the passive membrane time constant only by a small percentage. The energy cost of a high prevalence of highconductance states and extremely short membrane time constants would also exceed the energy budget of the brain (ref. 4). I would therefore suggest dropping this sentence.

We have clarified this sentence thanks to the reviewer's suggestion. We meant that the instantaneous, rather than the time-averaged, conductance can be very big. To clarify this we have rewritten this section (l. 15): This is especially relevant to integration of inputs during high conductance states that are prevalent in vivo, where a typical neuron receives significant synaptic drive. In these states, the effective membrane time constant can be extremely short, and varies substantially depending on synaptic input.

(3) l. 17f "As a consequence, computations that rely on passive summation of multiple inputs place punishing constraints on spike timing precision.": — Again, the passive membrane time constant is on the order of 10 ms and I would tone down this statement accordingly, removing the word 'punishing' for example.

Following the suggestion, we have rewritten the sentence to (l. 18): "As a consequence, computations that rely on passive summation of multiple inputs would place strong constraints on spike timing precision."

(4) l. 18ff "Dendritic action potentials, by contrast, have a consistently long duration that is ensured by the kinetic properties of voltage gated ion channels and NMDA receptors [54, 47, 10, 3]. These properties are largely determined by the amino acid sequence of receptor and channel proteins that are specifically expressed in dendrites [45, 44, 40]. This suggests dendritic properties are specifically tuned to produce localised, suprathreshold events that outlive rapid membrane fluctuations.": — Yes, but see also Attwell & Gibb 2005 (https://www.nature.com/articles/nrn1784), especially the last two of their key points. The slow NMDA receptor decay kinetics (and therefore their high affinity for binding glutamate) may also be the consequence of a design goal to set the temporal coherence window for NMDA receptor-mediated synaptic plasticity such as STDP to be on the order of tens of milliseconds, somewhat longer than the membrane time constant.

The reviewer is correct; other functions (e.g. synaptic plasticity) are also part of the dendrite's repertoire. To acknowledge this, we added a section (l. 34) where we mention that our idea does not conflict with, for example, synaptic plasticity.

(5) l. 32f "Numerous studies point out that nonlinear summation in dendrites can make neurons computationally equivalent to entire networks of simplified point models, or 'units' in a traditional neural network [9, 21, 38, 40, 45, 48, 50, 51].": — See also Beniaguev et al. 2021 (https://www.cell.com/neuron/pdf/S0896-6273(21)00501-8.pdf), which also speaks to the next sentence.

We thank the reviewer for the suggestion; the citation has been added.

(6) Fig. 2E and F: the top of panel F corresponds to the top of panel E, but the bottom ofpanel F does not correspond to the bottom of panel E - it corresponds to a dendritic neuron with passive dendrites, not a point neuron. Panel E should be changed to reflect this fact.

We have followed the suggestion to change the figure.

(7) l. 49f "Despite these dendritic spikes being initiated at different times, they still sum in the soma, leading to a sodium spike there (Figure 2E).": — You probably mean Fig. 2D, and instead of a sodium spike (which could be misunderstood as local and dendritic) you triggered a sodium action potential. Likewise, Fig. 2B (right) shows the timescale of sodium action potentials at the soma (cf. l. 46).

The error in the referencing to the figure has been corrected. The phrasing has also been changed to "a sodium action potential" (l. 56), following the reviewer's suggestion.

(8) Please check the scale bars in Fig. 2D. Do they also apply to panel F below? If yes thatshould be stated.

The scale bars are indeed the same; I have repeated them in the figure to avoid any confusion.

(9) l. 68 "This time constant is consistent with the high-conductance state of pyramidalneurons in the cortex [6]":

You do not need to invoke a high-conductance state to justify this time constant, which is indeed typical for the membrane time constant of pyramidal neurons in vivo.

On a related note, Fig. 3B and its legend seem to assume that tau = 1 ms, and calls that one EPSP duration in the legend. An EPSC may have a decay time constant of 1 ms, but an EPSP will have a decay time constant of about 10 ms, similar to the membrane time constant. Fig. 3B (and therefore also the rest of Figure 3) seems to have been constructed with a value of tau that is too small by a factor of 10, and this should be corrected by remaking the figure. If tau = 1 ms was used also in Figure 4 then this figure also needs to be remade.

Section 3.3 and Table 1 also use tau = 1 ms. This is unrealistic and needs to be changed an appropriate value of tau = 10 ms is given by the authors themselves in line 67. The incorrect value of tau in Table 1 causes other entries of the Table to be terribly wrong; a leak conductance of 1 µS would imply an input resistance of the neuron of 1 MOhm, but somatic input resistances of pyramidal neurons in vivo are on the order of 20 to 50 MOhm. The total capacitance of 1 nF is slightly too large, and should be adjusted to yield a membrane time constant of 10 ms given an appropriate leak conductance leading to an input resistance of about 20 to 50 MOhm. These are key numbers to get right for both Figures 3 and 4, especially if you want to be able to say "We have been careful to respect the essence of basic physiological facts while trying to build an abstraction of how elementary spiking computations might occur." (l. 215f).

We thank the reviewer for catching this. We had actually already used tau = 10 ms, but had not yet updated the paper. Moreover, the somatic input resistance was indeed off. To rectify this, we have used the values: $Cm = 0.5 nF$, $\taum = 10 ms$, $Rm = 20 M \Ohm$, $gl = 0.05 \mu S$. Figure 3 was remade using these values, and Table 1 updated accordingly.

(10) l. 158ff "The assumption that each neuron connects to one dendrite of an upstream neuron is actually grounded in physiology, although it may appear like a strong assumption at first glance: related inputs arrive at local clusters of spines synchronously [60].": — You probably mean "each neuron connects to one dendrite of a downstream neuron." And I would add "But see Beniaguev et al. 2022" - your restrictive arrangement of inputs is probably not really needed, especially if postsynaptic neurons have more dendrites.

The suggested wording was correct, and has been now incorporated (l. 166). I have also added the suggested citation.

(11) I note that the plateaus in Fig. 4D are much shorter than those in Fig. 2D and F, but thisis a good thing: The experimental and simulation results in Fig. 2 are based on ref. 18, which used microiontophoresis of glutamate, leading to much slower glutamate concentration time courses at the dendritic NMDA receptors than synaptic release of glutamate would. The time courses of plateaus in Fig. 4 are much more in line with the NMDA plateau durations shown in ref. 21, especially their Figure 2B. These faster NMDA plateaus (or NMDA spikes as they are called in ref. 21) are based on synaptic release of glutamate in vivo, and on the faster NMDA receptor kinetics at physiological temperature compared to the old models with room temperature kinetics used in ref. 18.

Here are two additional references that the authors might find interesting:

Fisek et al. 2023 https://www.nature.com/articles/s41586-023-06007-6 Dudai et al. 2022 https://www.jneurosci.org/content/42/7/1184.full

We thank the reviewer for the suggested references. The first has been added to the references in the introduction, on l. 28. The second has been added on l. 78.

**Reviewer #2 (Recommendations For The Authors):**

(1) In Fig. 3A, we observed some animal pictures, which were never explained in the figurecaption, or text of the manuscript. These pictures were probably explained at the lab meeting, so it is unnecessary to waste effort on these pictures in the manuscript draft.

We agree with the reviewer; the figures have been removed.

(2) Figure 1 has not been referenced anywhere in the manuscript text!

Indeed, this had to be corrected, figure is now references on l. 9.

(3) Line 45. "[18] triggered two NMDA spikes by glutamate uncaging at the indicated (red,blue) sites". [18] triggered one NMDA spike while recording at three locations simultaneously (two locations in dendrite and one location in the soma).

The reviewer is correct here. The sentence has now been rephrased to "(ref.) triggered an NMDA spike by glutamate microiontophoresis while recording at the soma and the indicated (red, blue) sites in the dendrite." (l. 49)

(4) Fig. 2B. The two labels, "Dendrite 2" and "Dendrite 1" incorrectly suggest that two traceswere recorded in two dendrites. These two traces were recorded in the same dendrite.

We agree with the reviewer; labels have now been changed to "Dendrite site".

(5) Line 45. "[18] triggered two NMDA spikes by glutamate uncaging at the indicated (red,blue) sites". - - One NMDA spike by "glutamate microiontophoresis".

This is correct, the phrasing on (l. 50) has been changed accordingly.

(6) Line 47. "... simulated glutamate releases 50 ms apart in the three dendritic sites indicatedin Figure 2C, thereby triggering three NMDA spikes at those sites. Despite these dendritic spikes being initiated at different times, they still sum in the soma, leading to a sodium spike there (Figure 2E)". A similar experiment has been performed in real cortical neurons (KD Oikonomu et al., 2012, PMID: 22934081), and could potentially be cited here. Briefly, Oikonomou et al. generated two dendritic plateau potentials in two dendritic branches and monitored the summation of these dendritic plateau potentials in the cell body.

The reference has been added, on l. 54

(7) Line 63. "We compared the behaviour of our simplified model with that of the full, detailedbiophysical model". Which detailed biophysical model? Please cite here the detailed biophysical model that you used for comparisons with your simplified abstract model.

The reference to the paper has been added.

(8) Line 65. "Figure 2F shows that spikes arriving at different times are summed in anintegrate and hold-like manner". In Fig. 2F, I am unable to see that spikes arriving at different times are summed in an integrate and hold-like manner. Which feature of Fig. 2F refers to the "hold-like manner"? Please explain in the manuscript.

To clarify we have added "Figure 2F, top" in the text (l. 71).

(9) Figure 2 caption. "(F) The voltage traces of the abstract model, with and without plateaus.Because of the extended time duration of the plateau potentials, they sum accurately to produce a somatic spike". I am unable to understand what an "accurate summation" in Fig. 2 is. Could the authors provide an illustrative example of a situation in which the neuronal potentials DID NOT sum accurately?

To address this confusion, we have changed the wording to "...they are summed to reach threshold."

(10) Line 75. "This is an important issue we intend to return to in future work". The authorspersonal plans should not be in the text discussing scientific results.

We believe it is entirely reasonable to discuss scientific plans that are part of ongoing work, and this is quite common throughout the literature. Nonetheless, we have now reworded this to "This is an important issue for future work." (l. 81)

(11) In Fig. 4F, the full-line and the dashed-line have not been identified! The readers are leftto guess.

This has now been addressed both with text inserts in the figure, and specification in the figure caption.

(12) Line 247. "would amount to scaling up the number of cells in a network to performcomputations that could, in principle, be performed by more robust single units". Did the authors mean to say: "would amount to scaling up the number of cells in a network to perform computations that could, in principle, be performed by a fewer (but more robust) single units"?

We have replaced the sentence with the reviewer's suggestion (l. 259)

(13) In the abstract, the authors repeat sentences: "the timescale of dendritic potentialsallows reliable integration of asynchronous inputs" and "nonlinear dendritic plateau potentials allow reliable integration of asynchronous spikes". Besides this being a bad writing style, the authors cannot decide if inputs to the model neuron are asynchronous, or spiking of the model neuron is asynchronous. Are these asynchronous spikes occurring in the neuron experiencing dendritic plateau potentials, or these asynchronous spikes occur in the neuronal network? This confusion of terms and ideas must be removed from the abstract.

We have rewritten the second sentence, which now reads: "Using this model, we show that long-lived, nonlinear dendritic plateau potentials allow neurons to spike reliably when confronted with asynchronous input spikes."

(14) In the abstract, the authors claim: "Our results provide empirically testable hypothesesfor the role of dendritic action potentials in cortical function". With great anticipation, I read throughout the manuscript, but I was unable to find one single experimental design that could support the authors' bald statement. In the text of the manuscript, the authors must carefully reveal the precise experimental outline that would test their specific hypothesis, or delete the untrue statement.

We respectfully challenge the rather critical tone of the reviewer. The central hypothesis that plateaus enable robust summation, and that circuit level computations rely on this is an experimentally testable hypothesis. The precise experimental design of how to test such a hypothesis is always best left to an experimentalist to determine, as there are many possible means of doing this and each will depend on the preparation and methodology at hand. At the same time, we understand that there is an increasing culture of expecting explicit "testable hypotheses" spelled out to the reader. To satisfy this expectation while avoiding overly prescriptive ideas for how future work should proceed, we have now added more explicit descriptions of possible experimental tests in l. 231 and onwards.

(15) Fig. 2F was submitted for review without a time scale, while at the same time the authorsheavily discuss specific numerical values for time intervals. Namely, the authors instruct readers to pay attention to a 10 ms time constant and 2-3 ms input decay (Fig. 2F), but they do not show the time scale in Fig. 2F.

"We compared this to a situation where all inputs arrive at a soma with standard LIF dynamics and a 10 ms membrane time constant. This time constant is consistent with the high-conductance state of pyramidal neurons in the cortex [6]: Inputs decay after 2-3 ms, and fail to sum to spike threshold (Figure 2F, lower)".

The time (and voltage) bars have now been added to Fig. 2F.

(16) Line 75. "In the scope of what remains here we want to ask if integrate-and-hold isminimally feasible". This reviewer is unable to understand the meaning of the syntaxes "integrate-and-hold" and "minimally feasible" in the context of dendritic integration. This reviewer is worried that the majority of the journal readers would feel exactly the same. To alleviate this problem, the authors should explain both terms right here, in line 77.

Integrate-and-hold is defined on line 57 to be exact we write: "We refer to this behavior as “Leaky Integrate-and-Hold” (LIH)." To be more clear we could reuse the acronym LIH here, to emphasise that we are referring to the same thing. By 'minimally feasible' we mean biologically plausible given assumptions that are not strong. Can use another term, e.g. "biologically plausible under lenient assumptions".

To address this point, we have rephrased the sentence as "In the scope of what remains here we want to ask if Leaky-Integrate-and-Hold (LIH) can easily and plausibly facilitate network computations with spikes." (l. 81), repeating the LIH definition.

(17) Line 91. "Spikes arriving even slightly out of sync with each other introduces noise in themembrane potential ..." Introduce.

The sentence has been fixed using the reviewer's correction.

(18) The caption of the Fig. 3B was submitted for review without any explanation of thenormalization procedure used. Also, in the caption of the same figure, one cannot find explanation of the light-gray area surrounding the black curves. Also, the readers are left to wonder how the results of a simulation could possibly be greater than 1 in some simulation trials.

We have added a description of the normalization and the shaded area to the caption of Fig. 3B.

(19) Line 117. "We assumed that inputs to a network arrive at the dendrites within some timewindow, and their combined depolarisations are either sufficient to either elicit a dendritic spike or not, as shown in Figure 3". We could potentially compact the current text by deleting one instance of "either".

We agree this is better writing; one of the occurrences of 'either' has been removed.

(20) Line 127. "where incoming connections can be represented with a 1 (a spike arrives)..."Did you mean "a presynaptic spike arrives"?

The sentence has been rewritten following the suggestion.

(21) Line 134. "with each unit only having ..." Dendrite can be a unit. Dendritic spine can be aunit. Did you mean "with each unit (i.e. neuron) having ..."

We have incorporated the suggestion.

(22) Fig. 4, Caption. "Each point is a 2D input vector x, the colors represent the differentclasses". An effort was made to introduce 3 different classes. But then, no mention of "classes" thereafter. The three input vectors, mentioned in Line 170, perhaps represent the remnants of the class concept mentioned in the previous paragraph.

We have now rewritten the sentence beginning with "These three input vectors ..." on l. 182 to emphasise that a correct answer means a correct classification.

(23) Line 152. "The 2D input points were first projected onto a binary feature space, to obtain13D binary vectors". Did you mean to say: "The 2D input points (three classes, Fig. A) were first projected onto a binary feature space, to obtain three binary vectors; each 13D binary vector responding to a specific class".

The sentence has been replaced with the reviewer's suggestion (l. 159).

(24) Line 163. "Because our focus is to account for how transient signals can be summed andthresholded robustly, we are assuming that inhibition is implicitly accounted for in the lumped abstraction". Could you please explain your two ideas: [1] "inhibition is implicitly accounted for" and [2] "lumped abstraction", because this reviewer did not get neither idea.

The reviewer is right that as it stood, the sentence was unclear. To clarify the point we have decided to expand upon that sentence and break it out as an individual paragraph (starting l. 171).
